# Methods of olfactory ensheathing cell harvesting from the olfactory mucosa in dogs

**DOI:** 10.1371/journal.pone.0213252

**Published:** 2019-03-06

**Authors:** Daisuke Ito, Darren Carwardine, Jon Prager, Liang Fong Wong, Masato Kitagawa, Nick Jeffery, Nicolas Granger

**Affiliations:** 1 Division of Regenerative Medicine, School of Clinical Sciences, University of Bristol, Bristol, United Kingdom; 2 Division of Veterinary Neurology, School of Veterinary Medicine, Nihon University, Fujisawa, Kanagawa, Japan; 3 Department of Small Animal Clinical Sciences, Texas A&M University, College Station, Texas, United States of America; 4 The Royal Veterinary College, University of London, Hawkshead Lane, Hatfield, Hertfordshire, United Kingdom & CVS referrals, Bristol Veterinary Specialists at Highcroft, Bristol, United Kingdom; Faculty of Animal Sciences and Food Engineering, University of São Paulo, BRAZIL

## Abstract

Olfactory ensheathing cells are thought to support regeneration and remyelination of damaged axons when transplanted into spinal cord injuries. Following transplantation, improved locomotion has been detected in many laboratory models and in dogs with naturally-occurring spinal cord injury; safety trials in humans have also been completed. For widespread clinical implementation, it will be necessary to derive large numbers of these cells from an accessible and, preferably, autologous, source making olfactory mucosa a good candidate. Here, we compared the yield of olfactory ensheathing cells from the olfactory mucosa using 3 different techniques: rhinotomy, frontal sinus keyhole approach and rhinoscopy. From canine clinical cases with spinal cord injury, 27 biopsies were obtained by rhinotomy, 7 by a keyhole approach and 1 with rhinoscopy. Biopsy *via* rhinoscopy was also tested in 13 cadavers and 7 living normal dogs. After 21 days of cell culture, the proportions and populations of p75-positive (presumed to be olfactory ensheathing) cells obtained by the keyhole approach and rhinoscopy were similar (~4.5 x 10^6^ p75-positive cells; ~70% of the total cell population), but fewer were obtained by frontal sinus rhinotomy. Cerebrospinal fluid rhinorrhea was observed in one dog and emphysema in 3 dogs following rhinotomy. Blepharitis occurred in one dog after the keyhole approach. All three biopsy methods appear to be safe for harvesting a suitable number of olfactory ensheathing cells from the olfactory mucosa for transplantation within the spinal cord but each technique has specific advantages and drawbacks.

## Introduction

Olfactory ensheathing cells, also known as olfactory glial cells, are found in the olfactory mucosa and olfactory bulb of mammals, and support axonal regeneration of olfactory sensory neurons throughout life [1–6]. In the normal olfactory system, olfactory ensheathing cells are able to guide newly growing olfactory nerve axons from the olfactory mucosa to the olfactory bulb, and interact with astrocytes at the level of the boundary with the olfactory bulb in the central nervous system (CNS). When transplanted, they can ensheath and myelinate regenerating axons in the spinal cord [7–9].

In view of these axon growth-promoting properties, olfactory ensheathing cell transplantation is a promising strategy for spinal cord repair following spinal cord injury (SCI). Although disrupted axons often sprout and regrow after SCI they fail to reach their targets on the other side of the lesion because of the inhibitory environment they face. This includes inflammatory mediators, the glial scar that contains axon growth-inhibiting factors and cystic cavities in the lesion [10–12]. It is thought that olfactory ensheathing cells might guide, support and myelinate regenerating axons as they grow through damaged regions of the CNS because of their ability to modulate immune responses [13–15], provide neurotrophic factors [16], remyelinate demyelinated axons [17,18], modulate glial and neuronal function [14] and as neuroprotective agents [15]. Indeed, many studies on olfactory ensheathing cell transplantation in experimental SCI animal models have demonstrated their efficacy in spinal cord regeneration, both histopathologically and functionally [9,19,20].

When selecting a source for transplanted olfactory ensheathing cells an autologous source is highly attractive since it avoids the need for a donor and the need for immunosuppression after transplantation, which, although it improves the survival of allogenic transplants, can carry risks of its own [21–24,25]. Olfactory ensheathing cells can be obtained either from the olfactory bulb (central olfactory ensheathing cells) or from the olfactory mucosa lining the nasal cavity and frontal sinus (peripheral olfactory ensheathing cells) [26–31]. For practical application the mucosal source is preferable because it avoids the requirement for craniotomy. It has already been found that biopsy of the olfactory bulb is associated with a risk of adverse events in dogs: 10% of dogs undergoing olfactory bulb biopsy in one study developed late-onset seizures [32]. Furthermore, the olfactory bulb is not an ideal source of autologous olfactory ensheathing cells in humans because it is small and relatively inaccessible. Instead, the olfactory mucosa can be obtained by minimally-invasive methods such as rhinoscopy in humans [26,33].

For these practical reasons, although it has been documented that ‘peripheral olfactory ensheathing cells’ and ‘central olfactory ensheathing cells’ might have different regeneration-generating potential *in vivo* [34,35], the focus in translational medicine has been on mucosal-derived cells, especially since it has been established that human and rodent mucosal olfactory ensheathing cells promote axonal sparing [36,37] and ameliorate neurological functions after laboratory SCI [36,38]. Furthermore, clinical trials of mucosal-derived olfactory ensheathing cell transplants have been performed in both species [25,32,39–46].

Here our objective was to describe and compare three methods of collecting olfactory ensheathing cells from the olfactory mucosa to provide information on the limitations and advantages of different methods.

## Material and methods

### Ethical considerations

The surgical and endoscopic procedures described in this report were all conducted with ethical approval from relevant ethical committees at each institution [*i*.*e*. NG/NDJ: for cases recruited in Cambridge, the procedures were reviewed and approved in September 2008 by the Royal College of Veterinary Surgeons and the Ethical Review Committee of the Department of Veterinary Medicine, University of Cambridge, where the study was carried out (there was no number given to the ethical application for this project at the time); NG: for cases recruited in Bristol, the Local Ethical Committee granted the number VIN/13/033 in June 2013; and DI: the Ethical number was NUVM225 and the research was approved in October 2009 by the Nihon University Ethical Committee for cadaver dogs and cases recruited at Nihon University].

### Recruitment of dogs and owner’s informed consent

Clinical cases with spinal cord injury were all client-owned dogs (*i*.*e*. companion animals) that had lived mainly in the UK, although some originated from European countries. Dogs were recruited by two authors (NG, NDJ) using email advertisement to UK veterinary referral hospitals, press releases from the University of Cambridge, articles in the veterinary press and oral and poster communications such as during dog shows in the UK or veterinary conferences. These dogs were part of clinical trials and we have previously published data showing the efficacy of the cell transplants that were obtained [40]. After we were contacted by their owners, dogs were examined for inclusion in the clinical trial. For the samples obtained from normal (living) dogs, we asked consent from owners of dogs undergoing bronchoscopy or rhinoscopy for other reasons (*i*.*e*. bronchitis, foreign body) for inclusion of their dogs in this study (DI).

In all cases, consent was obtained from each dog’s owner after a first consultation explaining the work to be carried out during our clinical trials, and the risks and possible benefits for the animals. The consent was recorded on a paper form signed by the owners of the dog and all consent forms were stored in a folder or with medical records.

### Surgical procedures

All surgical procedures were performed under general anaesthesia using either isoflurane or sevoflurane, and all efforts were made to minimize pain after surgery (see also statement of post-operative pain management).

#### Frontal sinus rhinotomy

**Fig 1:**
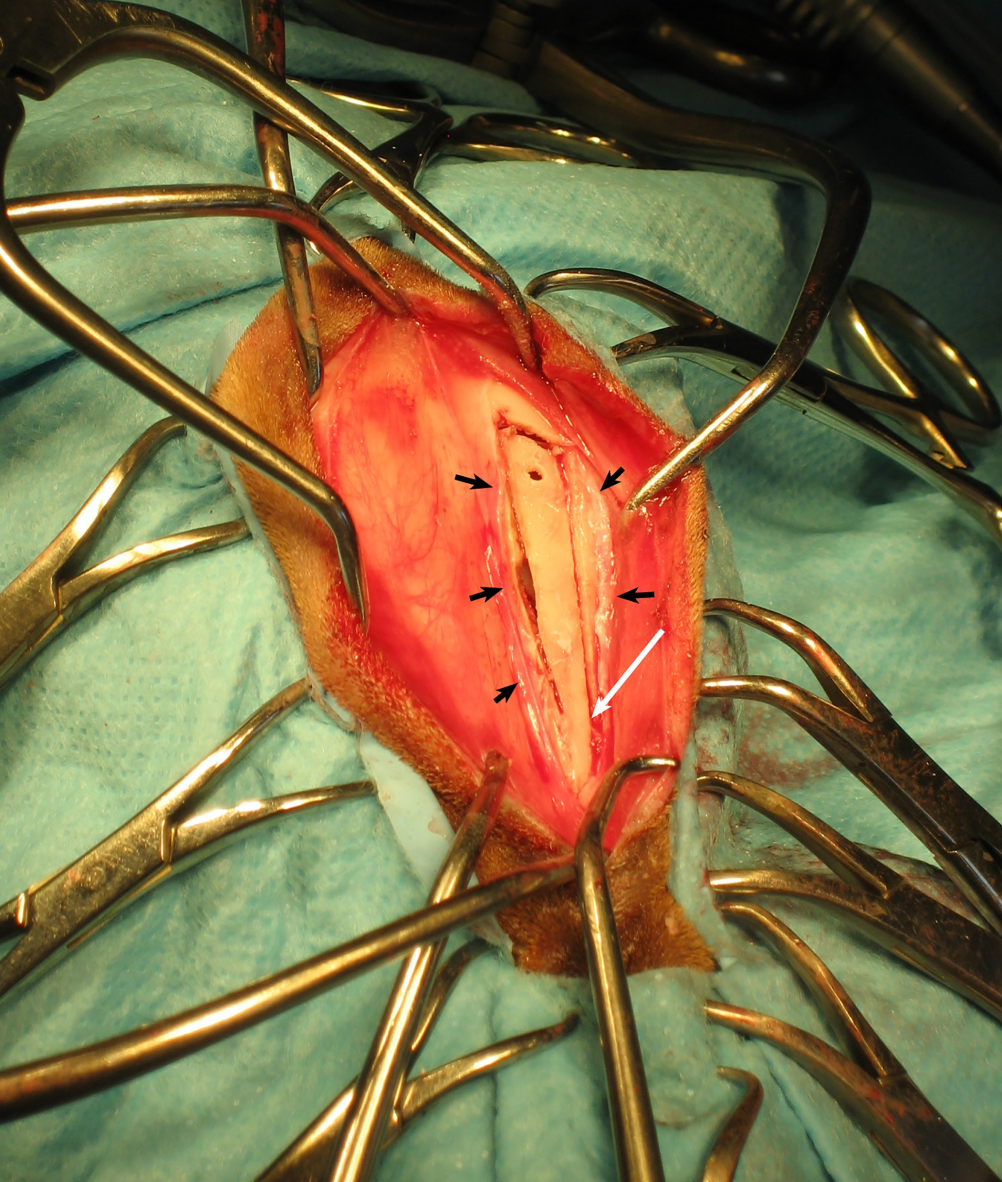
Dorsal approach of the left frontal sinus for olfactory mucosa harvesting and later spinal cord transplantation, in a dog with chronic paralysis. The nasal and frontal bones are cut above the nasal cavity and frontal sinus to form a bone flap. The periosteal layer (black arrows) is preserved to be sutured at the end of the procedure. The rostral aspect of the bone flap is kept intact (white arrow). A hole (visible on the cranial aspect of the bone flap) has been made using surgical drill to suture the bone flap back into position at the end of the procedure.

**Fig 2:**
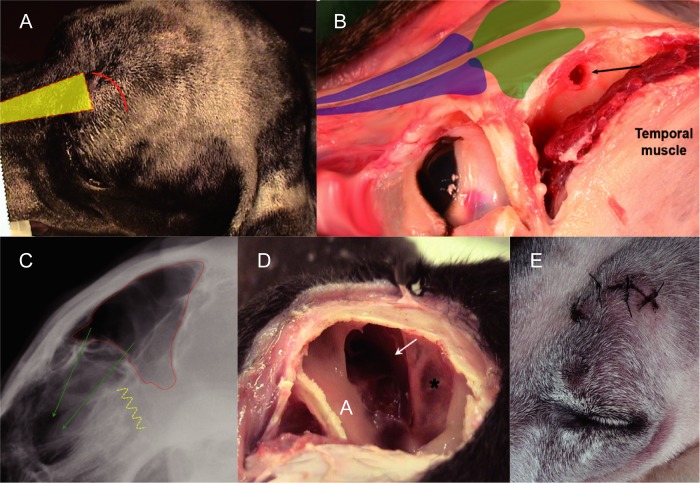
Surgical approaches to the frontal sinus. (**A**) frontal sinus region of a dog prepared for surgery; the frontal sinus can be approached caudo-laterally through an incision above the left eye (red curved line) and then through the temporal bone, or more classically along the nose midline through the nasal bone (yellow shape and dashed red-line–see Fig 1 as well); (**B**) lateral view of a dog cadaver showing the incised left temporal muscle detached and elevated from the temporal bone to expose the cranial part of the temporal bone that forms the caudal wall of the frontal sinus (location suggested by the green shapes); a circular bony window (black arrow) was drilled in the caudal wall of the frontal sinus to access it; the window allowed the insertion of biopsy instruments (alligator or long curved forceps); the blue shapes represent the nasal cavities; (**C**) lateral radiograph showing the anatomical relationship between the frontal sinus (red dotted line), the nasal cavities (green arrows) and the cribriform plate (yellow dotted curved line) just cranial to the brain; (**D**) caudal view of a dog cadaver with the left frontal sinus open, showing the difference between the pink pale respiratory mucosa (*) and the brown olfactory mucosa (white arrow) deeper in the cranial aspect of the frontal sinus and communicating through the ostium with the nasal cavity; (**E**) 5-day post-operative view of a dorso-lateral incision above the left eye made to collect olfactory mucosa from the frontal sinus in a paraplegic dog, weighing > 10 kg.

The skin was incised along the nasal midline and the periosteum lying above the left nasal and frontal bone was incised and elevated but preserved. The nasal and frontal cortical bones were cut with a surgical oscillating saw progressing in a sagittal orientation from the caudal half of the nasal bone towards the frontal bone adjacent to the midline, then around the rostral margins of the left frontal sinus and, finally, the lateral part of those bones on the left hand side of the nasal cavity and frontal sinus (Figs 1 and 2A–2C). The rostral attachment of the nasal bone was preserved, leaving a fixed bone flap that was elevated to access the left nasal cavity and frontal sinus. The frontal sinus mucosa was identified at the junction of the nasal cavity and the frontal sinus (adjacent to the ostium) by its characteristic brownish colour (contrasting with the pink colour of the respiratory mucosa) (Fig 2D). The olfactory mucosal biopsy was obtained by carefully inserting artery forceps into the ostium of the passage between the frontal sinus and the nasal cavity. The bone flap was sutured back into place using burr holes pre-drilled in the bone flap (Fig 1) and the undisturbed bone overlying the frontal sinus. The periosteal layer was sutured to form an additional layer of bone coverage.

#### Keyhole approach

This surgical approach was developed to access one frontal sinus caudally *via* a small temporal bone incision (see description of the technique in Fig 2). Briefly, the skin incision was made above the upper eyelid and overlying the frontal bone (Fig 2A), followed by incision of the temporal muscle fascia, taking care to leave enough fascia on the bone side for purchase for suturing. The muscle was elevated from the temporal bone to expose the cranial part of the temporal bone that forms the caudal wall of the frontal sinus (Fig 2B). A circular bony window was drilled in the caudal wall of the frontal sinus. Biopsy instruments (alligator or long curved forceps) were inserted into the frontal sinus through the window. The olfactory mucosa was grasped blindly and three pieces of the mucosa were obtained. The bone window was left open but sealed by the temporal muscle reflected back against the temporal bone. The temporal muscle fascia and skin were sutured routinely (Fig 2E).

#### Endoscopic procedures using flexible endoscopy

**Fig 3:**
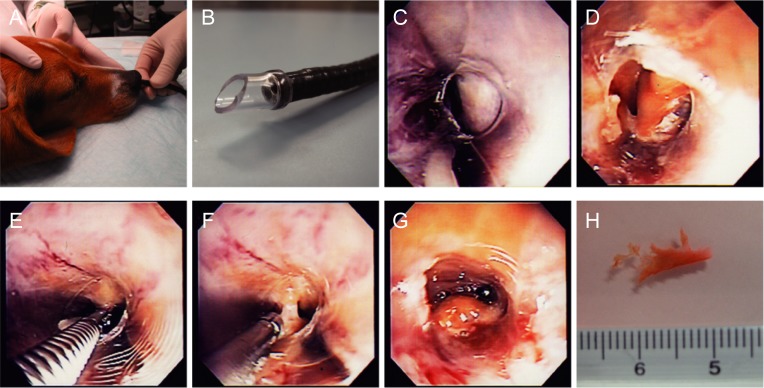
Endoscopic approach to the olfactory mucosa *via* the natural nasal passage. (**A**) dogs were positioned in sternal recumbency and one side of the nasal passages was washed with sterile saline containing gentamicin; (**B**) silicon tube attached to the end of the endoscope. The space inside the tube kept the vision clear and allowed clean biopsy of olfactory mucosa; (**C**) pale-coloured respiratory mucosa could initially be seen; (**D**) brown-coloured olfactory mucosa could be observed more caudally in the nasal cavity; (**E**)(**F**) harvesting olfactory mucosa using endoscopic biopsy forceps without damaging other structures; (**G**) bleeding after biopsy; (**H**) fragments of olfactory mucosa biopsied and placed in L15 medium.

This technique was initially investigated in fresh cadaver dogs to determine feasibility and then applied in live dogs. With the dog under general anesthesia the nasal cavity of either side (left or right were randomly selected) was washed three times with sterile saline containing gentamicin (10mg/mL). Either a 3.8mm or 5.0mm diameter Olympus VQ5112 endoscopic probe (depending on the size of the dog) was inserted into the nasal passage (Fig 3A). A short piece of sterile silicone-plastic tube was added to the probe tip to make a space between the structures in the nasal passage and the tip of the probe to permit clear vision and obtain a clean biopsy (Fig 3B). Initially, pale respiratory mucosa was apparent in the rostral nasal passage (Fig 3C), then the brown olfactory mucosa was found in the caudal cavity (Fig 3D–3G). Once olfactory mucosa was located, sterile endoscopic biopsy forceps were inserted to harvest the mucosa. The olfactory mucosa was gently peeled from the turbinate bone, keeping the bone intact (Fig 3E–3G). Four pieces of olfactory mucosa were obtained from each dog and three pieces of olfactory mucosa were prepared for cell cultures and the remaining piece prepared for immunohistochemistry.

### Post-operative pain management

For surgical procedures, dogs received pre- and post-operative (24 hours) methadone (0.3mg/kg IM every 4 hours) and a non-steroidal anti-inflammatory drug for 5 days (carprofen, 2mg/kg/day *per os*). For endoscopic procedure, dogs received pre-operative mixture of analgesia containing opioid (butorphanol, 0.2mg/kg SC) and a benzodiazepine (midazolam, 0.2mg/kg SC); a non-steroidal anti-inflammatory drug (carprofen, 2mg/kg/day *per os*) was administered post-operatively for 2 days.

### Cell culture methods

Cells were cultured according to previously reported methods, with modification [30]. Briefly, blood vessels, connective tissues and bone (if included) were removed under a dissecting microscope and the tissues were chopped with a scalpel blade. The tissue was dissociated in 0.5mL of collagenase solution (6.66mg/mL in L-15; 155U/mg, MP biomedical, Heidelberg, Germany) for 20 minutes at 37°C in 5% CO_2_. 0.5mL trypsin solution (2.5mg/mL, 10,000 BAEE, Sigma-Aldrich Co Ltd, Shaftesbury, Dorset, UK) was added and the mixture kept at 37°C in 5% CO_2_ for another 15 minutes. After the incubation the tissue was rinsed in 10mL Dulbecco modified Eagle medium (DMEM) with 10% foetal bovine serum (Sigma-Aldrich Co Ltd, Shaftesbury, Dorset, UK), termed ‘olfactory mucosal cell basic medium’ (OMBM), and centrifuged at 1000rpm (157 x g) for 7 minutes. The pellet was re-suspended in 1mL of soybean trypsin inhibitor (0.25 mg/mL, Sigma-Aldrich Co Ltd, Shaftesbury, Dorset, UK) and bovine pancreas DNAase (0.04mg/mL, Sigma-Aldrich Co Ltd, Shaftesbury, Dorset, UK) solution with bovine serum albumin (3mg/mL, Sigma-Aldrich Co Ltd, Shaftesbury, Dorset, UK) and triturated 10 times through a 5-mL plastic pipette and once each through a 21- and 23-gauge needle with a 1mL plastic syringe. The processed tissue was washed with 10mL of OMBM and centrifuged. The pellet was re-suspended in serum-free medium containing DMEM, 2μM forskolin (Sigma-Aldrich Co Ltd, Shaftesbury, Dorset, UK) and 20ng/mL neuregulin-1 (R&D systems, Abingdon, Oxon, UK), and seeded (50,000 cells/mL) into a poly-L-lysine (Sigma-Aldrich Co Ltd, Shaftesbury, Dorset, UK) coated 25cm^2^ flasks. Cultures were maintained at 37°C in 5% CO_2_ and one half of the medium was renewed every 3 days. After culturing for 7 days, the culture medium was replaced by OMBM with forskolin and neuregulin-1 at same concentration, here defined as ‘growth medium’ (GM). The GM was renewed every 3 days. On occasions, antibiotics were used such as penicillin / streptomycin and then gentamicin if an infection was suspected. Once the cells became confluent they were passaged to new poly-L-lysine coated flasks. At 21 days *in vitro*, cells were passaged to either glass cover slips in 24-well plates or 8-chamber Lab-Tek counting slides (Nalge Nunc International, Rochester, NY, USA) from each flask and kept in 500μL (cover slips) or 250μl (chamber slides) of GM at 37°C for 24 hours. After incubation, the cells were fixed with 4% buffered paraformaldehyde and maintained in PBS solution at 4°C until immunostaining.

### Immunofluorescent studies

For blocking, slides were incubated in 10% normal goat serum diluted in PBS for 1 hour at 21°C. After blocking, slides were double stained using the following primary antibodies: low-affinity mouse nerve growth factor receptor (p75, MAB5264, Millipore, Germany) and polyclonal rabbit anti-human fibronectin (Fn, A024502, Dako Cytomation, Cambridgeshire, UK) in 1:100 and 1:400 concentrations respectively, applied to cells obtained using the different biopsy methods and incubated for 45 minutes at 21°C. In addition, for cells obtained by rhinoscopy, which was the most recent technique of three methods we used, double-staining with p75 and polyclonal rabbit anti-glial fibrillary acidic protein (GFAP, Z0334, Dako Cytomation, Cambridgeshire, UK) at 1:100 concentrations were performed using the same incubation time. Slides/cover slips were washed with PBS solution 3 times for 5 minutes and secondary antibodies [goat anti-rabbit fluorescein isothiocyanate-conjugated (FITC, Southern Biotech, Birmingham, UK), and goat anti-mouse Cy3-conjugated (Jackson Immuno Research, West Grove Pa.)] were applied at a concentration of 1:200 for 45 minutes at 21°C. All antibodies were diluted in PBS solution. After incubation with secondary antibodies, slides/cover slips were washed with PBS solution 3 times for 5 minutes and mounted with 4’, 6-diamidino-2-phenylindole-containing mounting medium (DAPI, Vector Laboratories, Orton Southgate, Peterborough, UK). In this study, cells obtained by rhinotomy and keyhole approach were double-stained for p75 and Fn, and cells obtained by rhinoscopy were double-stained for p75 and Fn, and p75 and GFAP.

For immunofluorescent examination of mucosal tissues, samples obtained by rhinoscopy were immersed in 4% buffered paraformaldehyde for 24 hours then placed into 30% buffered sucrose for 48 hours. The tissues were then embedded in OCT and 12μm sections were cut using a cryostat. All sections were kept at -80°C until immunostaining. The same procedure as for the immunocytochemistry was performed with modified incubation times and temperatures. Primary antibodies were incubated overnight at 4°C and secondary antibodies were incubated for 1 hour at room temperature.

### Data collection

The cells were counted in a haemocytometer at 21 days *in vitro*. The proportion of immunolabelled cells in each condition was determined from a count made in at least five fields (at least 100 cells in each field) using x20 objective and fluorescent filter.

### Statistical analysis

In each cell culture obtained using the three different methods, the correlation between olfactory ensheathing cell yield (proportion and population) with age and body weight of dogs were assessed using the Spearman rank correlation coefficient. In addition, the Kruskal-Wallis test was used to compare proportion and population of olfactory ensheathing cells between the three methods; *post hoc* pairwise comparisons using Dunn’s test were applied if the original test indicated significant differences among the methods. For cells obtained by rhinoscopy, the Mann-Whitney test was used to compare proportion and population of olfactory ensheathing cells between cadaver and living dogs. A value of p<0.05 was taken to indicate statistical significance. All statistical analysis were performed using GraphPad Prism 5.01 (GraphPad Software Inc, La Jolla, California, USA).

## Results

### Demographics

Twenty-seven dogs with chronic severe SCI had olfactory mucosa harvested *via* frontal sinus rhinotomy. Their mean body weight was 6.97kg (+/- 2.19kg), and ages ranged between 4 and 14 years (mean 6.04 +/- 2.36 years). The supra-orbital keyhole approach to the frontal sinus was used in 7 dogs with chronic severe SCI that had a mean body weight of 17.8kg (+/- 6.53kg), and ages ranged between 2 and 10 years old (mean 6.00 +/- 2.65 years). Rhinoscopy was used in 13 cadaver dogs and 8 living dogs (these comprised of one dog with chronic severe SCI and 7 live dogs without nervous system disease). The body weight of dogs undergoing rhinoscopy (including cadavers) ranged between 2.1 and 13.5 kg (mean 6.56 +/- 3.42kg) and ages ranged between 2 to 14 years (mean 6.33 +/- 2.54 years). The dog breeds included in this study are summarized in Table 1.

**Table 1:** Dog breeds included in the three different methods.

Methods of biopsy (number of dogs)	Dog Breed (number of dogs)
**Rhinotomy (27)**	**<10kg**: Dachshund (20), Cavalier King Charles Spaniel (1), Jack Russel Terrier (1), Yorkshire Terrier (1), Terrier cross breed (1), Patterdale Terrier (1)
	**>10kg**: Dachshund (1), Welsh Corgi (1)
**Keyhole approach (7)**	**<10kg**: Dachshund (1)
	**>10kg**: Staffordshire Bull Terrier (2), Border Collie (1), English Cocker Spaniel (1), Irish Setter (1), Siberian Husky (1)
**Rhinoscopy (21)**	**Cadaver dogs (13)** **<10kg:** Beagle (6), Miniature Dachshund (2), Mixed Breed (1) **>10kg**: Beagle (3), Golden Retriever (1)
	**Living dogs (8)** **<10kg**: Miniature Dachshund (4), Shiba dog (1), Jack Russel Terrier (1), Mixed breed (1), Yorkshire terrier (1) **>10kg**: None

### Cell culture results

Olfactory mucosal cell cultures obtained from one cadaver and one live dog using rhinoscopy had uncontrollable bacterial infections at 9 and 3 days *in vitro* respectively, and all the cells died. Bacterial culture revealed *Escherichia coli* and *Streptococcus spp* in the culture medium from the cadaver dog, and *Streptococcus aureus* in that from the live dog.

**Fig 4:**
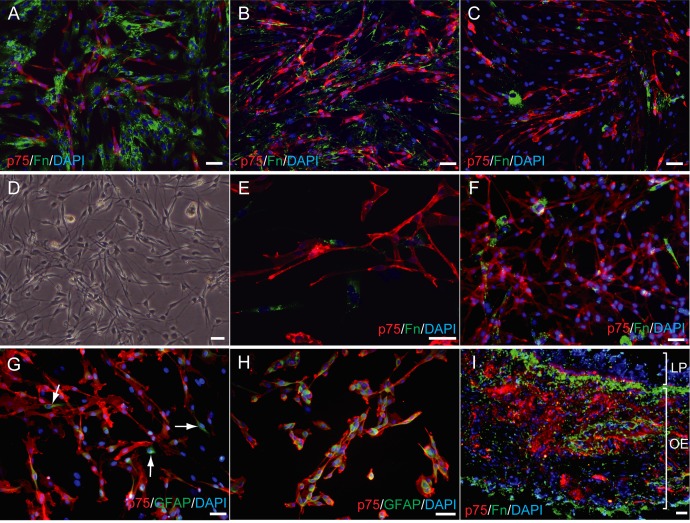
Immunocytochemical and morphological characteristics of olfactory mucosa cell cultures obtained from three different methods. All images are from *in vitro* cell cultures except image **I** obtained from an olfactory mucosa biopsy. Upper layer (**A**)(**B**)(**C**): cells obtained *via* rhinotomy; middle layer (**D**)(**E**): cells obtained *via* keyhole approach; and right middle and bottom layer (**F**)(**G**)(**H**): cells obtained *via* endoscopy; (**I**) immunohistochemistry of olfactory mucosa. Immunocytochemical characteristics of olfactory mucosa cell cultures obtained by rhinotomy in three dogs, showing variation in p75 positive cell (red) purity (**A**)(**B**)(**C**); images show a purity of ~25% (**A**), of ~50% (**B**) and of ~75% (**C**). Morphological characteristics of olfactory mucosa cells obtained by keyhole approach in bright light (**D**): note the spindle-shaped bipolar cells that form the majority of cells. Olfactory mucosa cell cultures obtained by the keyhole approach which are double stained with p75 and fibronectin (**E**): spindle-shaped bipolar cells expressed p75. The p75-positive cells obtained by endoscopy were morphologically and immunocytochemically identical to the p75-positive cells obtained using the other two methods (**F**)(**G**)(**H**). Most of the p75 positive cells were also positive for GFAP (**G**)(**H**). In rare instances, other cells with a bipolar and elongated morphology were solely GFAP-positive (white arrows in **G**). Immunofluorescence of the olfactory mucosa showed p75-positive area (cells) in the olfactory epithelium (OE) layer (**I**). LP: *lamina propria*. Bar = 50μm.

Generally, the morphology and immunostaining characteristics of the cell populations at 21 days *in vitro* obtained using the three methods were similar. The p75-positive cells, considered to be olfactory ensheathing cells, appeared elongated, bipolar or multipolar, spindle-shaped, and some of the p75-positive cells also expressed Fn (Fig 4). Most of the p75-positive cells obtained by rhinotomy co-expressed GFAP (Fig 4F and 4H). There were Fn-positive cells which did not stain with other antibodies and these were flat and stubby (Fig 4A, 4B and 4C). Cells labelling only with GFAP varied greatly in morphology ranging from stubby to bipolar (Fig 4F). Histologically, p75-positive cells were observed in the *lamina propria* of the olfactory mucosa (Fig 4I).

**Fig 5:**
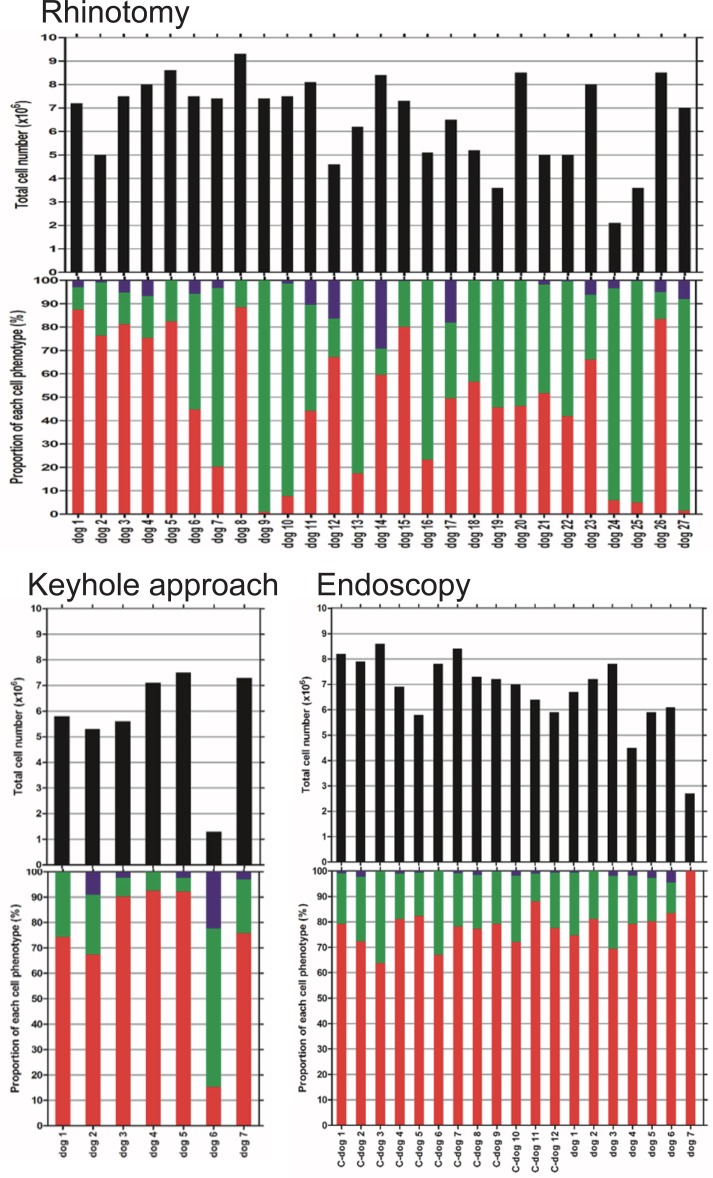
Proportion and population of each phenotype of cells obtained by three different methods. Upper histogram: number of cells (black). Lower histogram: corresponding proportion (%) of p75-positive cells (in red), fibronectin positive cells (in green) and unidentified cells (in blue) for each dog in different method.

**Fig 6:**
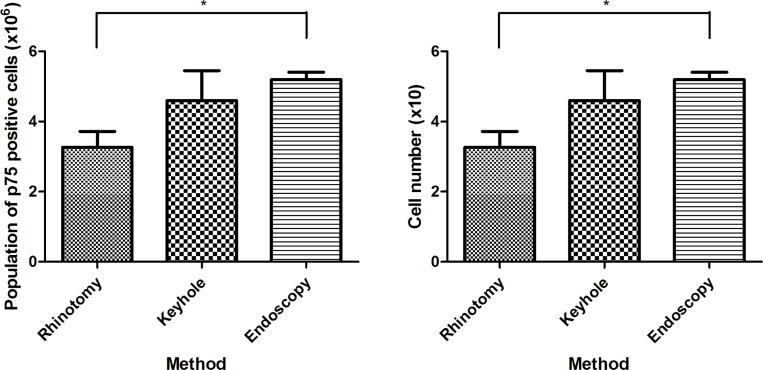
**Comparison of proportion (left) and population (right) of p75 positive cells in between different harvesting methods.** There were significant differences in p75-positive cell proportion and population in between cultures obtained by rhinotomy and endoscopy (P<0.01, **; P<0.05, *).

**Fig 7:**
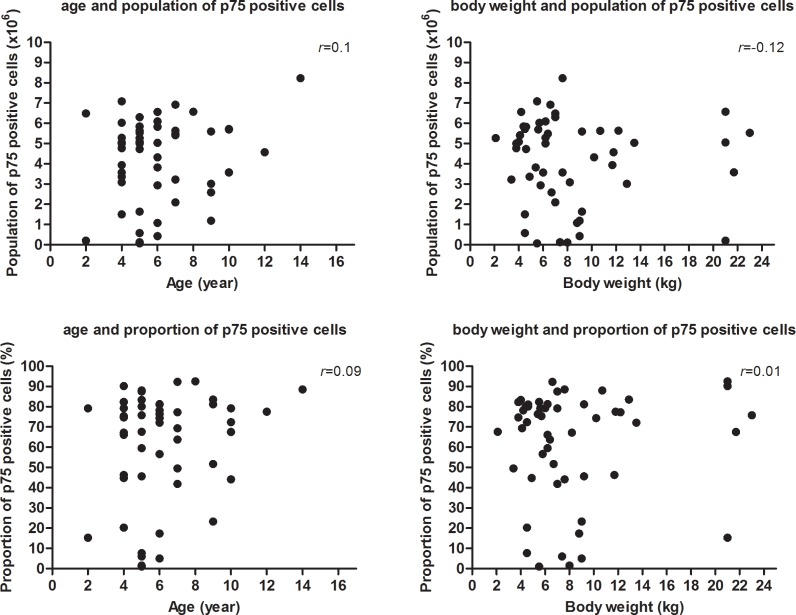
Correlation between olfactory ensheathing cell yield (population and proportion) and age, or olfactory ensheathing cell yield (population and proportion) and body weight. No correlation was observed.

The proportions and populations of each cell phenotype in different cultures are summarized in Fig 5, Tables 2 and 3 (details of cell population and proportion in cell cultures obtained by rhinotomy from 21 Dachshunds are available in S1 Appendix). The proportions and populations of p75-positive cells obtained by the keyhole approach and rhinoscopy were similar, representing approximately 70% of the total number of cells, *i*.*e*. ~4.6 x 10^6^ cells. However, fewer were obtained by frontal sinus rhinotomy; the proportion was ~48% and the population and was ~3.2 x 10^6^. Statistical analysis revealed that there were significant differences of proportion and population of olfactory ensheathing cell between the cells obtained by rhinotomy and endoscopy; p = 0.002 and 0.015 respectively (see Fig 6, Tables 2 and 3 for mean values). There was no correlation observed between olfactory ensheathing cell yield and age, or olfactory ensheathing cell yield and body weight when results from all methods were pooled (Fig 7). When comparing proportion and population of olfactory ensheathing cells obtained by rhinoscopy between cadaver and live dogs (including one dog with spinal cord injury), no significant difference was detected (P = 0.253 and 0.069 respectively).

**Table 2:** Summary of Cell Proportion and Phenotypic Characteristics in Cultures Obtained by Rhinotomy (*n* = 27), Keyhole approach (*n* = 7) and Rhinoscopy (Cadaver *n* = 12, Living dog *n* = 7) at 21 Days *In Vitro*.

	Proportion of cells for each Immunostaining Marker		
Method	p75+	Fn+	unidentified/*GFAP+
**Rhinotomy**	48.54 +/- 5.63	46.81 +/- 5.95	4.64 +/- 1.33
**Keyhole**	72.57 +/- 10.25	21.83 +/- 7.48	5.61 +/- 3.02
**Rhinoscopy**			
- All dogs	78.25 +/- 1.81	20.62 +/- 1.84	1.17 +/- 0.26
- Cadaver	76.5 +/- 1.94	22.9 +/- 1.98	0.69 +/- 0.20
- Case	81.14 +/- 3.60	17.23 +/- 3.51	1.63 +/- 0.61

Data represent the mean ± SEM percentage (%) labeled with each antibody. p75: low-affinity mouse nerve growth factor receptor; Fn: polyclonal rabbit anti-human fibronectin.

*GFAP: polyclonal rabbit anti-glial fibrillary acidic protein. *GFAP was applied to cells obtained by rhinoscopy; non-Fn+, non-p75+ cells obtained by the other two methods are described as unidentified.

**Table 3:** Summary of Estimated Cell Population and Phenotypic Characteristics in Cultures Obtained by Rhinotomy (*n* = 27), Keyhole approach (*n* = 7) and Rhinoscopy (Cadaver *n* = 12, Living dog *n* = 7) at 21 Days *In Vitro*.

	Cell numbers for each Immunostaining Marker			
Method	p75+	Fn+	unidentified/GFAP+*	Total
**Rhinotomy**	3.27 +/- 0.45	3.01 +/- 0.43	0.32 +/- 0.10	6.60 +/- 0.35
**Keyhole**	4.60 +/- 0.86	0.92 +/- 0.19	0.19 +/- 0.06	5.70 +/- 0.81
**Rhinoscopy**				
- All dogs	5.20 +/- 0.21	1.48 +/- 0.17	0.08 +/- 0.02	6.75 +/- 0.33
- Cadaver	5.54 +/- 0.17	1.68 +/- 0.19	0.07 +/- 0.19	7.28 +/- 0.27
- Case	4.62 +/- 0.42	1.13 +/- 0.27	0.10 +/- 0.04	5.84 +/- 0.66

Data represent the mean ± SEM × 10^6^ number of cells labeled with each antibody. p75: low-affinity mouse nerve growth factor receptor; Fn: polyclonal rabbit anti-human fibronectin. GFAP was applied to cells obtained by rhinoscopy; non-Fn+, non-p75+ cells obtained by the other two methods are described as unidentified.

*GFAP: polyclonal rabbit anti-glial fibrillary acidic protein.

### Adverse effects

After biopsy of olfactory mucosa by frontal sinus rhinotomy, three of 27 dogs showed emphysema and one dog (the smallest dog of our cohort, weighting 3.4kg) showed cerebrospinal fluid (CSF) rhinorrhea. This CSF rhinorrhea was caused by a cribriform plate fracture visible on computed tomography and continued for ~1 year. It was decided to avoid surgical intervention to correct the fractured cribriform plate in this very small dog and the dog remained in good health with very mild discharge but no adverse effect noted. All dogs had a small amount of epistaxis that disappeared within 24 hours of surgery.

In all dogs in which the keyhole approach was used, there was a small amount of epistaxis that ceased within 24 hours of surgery. One dog showed blepharitis which also quickly resolved in 24 hours.

Following rhinoscopy there was moderate bleeding immediately after taking the samples but this stopped within a few minutes without any specific treatment (Fig 3G). Three of eight dogs exhibited sneezing and a mildly bloody nasal discharge after recovery of anaesthesia, but this ceased by the following day.

There was no change in appetite following any of the biopsy methods and none of the dogs required any specific medical attention.

## Discussion

All three biopsy methods appear to be safe for harvesting mucosal olfactory ensheathing cells from dogs. Each provides a sufficiently large population of appropriate purity p75-positive cells for transplantation, but there are specific advantages and drawbacks associated with each technique.

Rhinotomy is the most invasive technique and incurs the risk that the bone flap might detach during or after surgery, although this has not been a problem in our hands. Even if it were to occur this would lead to little risk of poor bone healing, providing that the periosteal layer is preserved and sutured adjacent to the bone flap [47]. We observed subcutaneous emphysema in three dogs and CSF rhinorrhea in one dog as adverse effects. Emphysema, which is often observed in dogs undergoing rhinotomy [48], gradually resolved during the week following biopsy. However, from a cosmetic point of view, emphysema would not be ideal for owners, along with a relatively long longitudinal skin incision along the nose. One dog developed CSF rhinorrhea following the frontal sinus biopsy, which is a complication rate (3.7%) similar to that reported following frontal sinus surgery in human patients (6.5%) [49] and may carry a risk of infection of the central nervous system [50,51].

The keyhole approach comes with fewer of these risks. Although one dog showed blepharitis, it resolved quickly without specific treatment. However, the method was more difficult to perform in smaller and brachycephalic dogs, which may have small or non-existent frontal sinuses, thereby making this technique impossible. We would therefore recommend it for mesaticephalic or dolicocephalic dogs weighing more than 10 kg. This approach has the advantage of creating a minimal bony defect compared to the classical approach through the nasal bone, providing a better post-operative cosmetic result. This approach also limits the risk of post-operative subcutaneous emphysema because the bony defect created in the temporal bone is covered by the large mass of temporal muscle. The risk of damaging the cribriform plate is also avoided.

Rhinoscopy is the least invasive technique, with only a little epistaxis after biopsy and no skin incision. In addition, the populations and proportions of p75-positive cells were similar to those obtained by other methods. However, the limitation of this technique appears to be an increased risk of infection in the cell cultures; there were uncontrollable infections in cell cultures from two of 21 dogs (one from a cadaver and one from a live animal). There are many species of bacteria, including *Escherichia coli* and *Streptococcus spp*, in the nasal passage of healthy dogs [52–54]. Furthermore, in contrast to the other two methods, the endoscopy instrument cannot be totally sterilized, although it was washed and disinfected according to manufacturer guidance before use.

The three methods each yielded a sufficient number of cells for transplantation in most dogs, which has been estimated to be approximately 5 x 10^6^ cells from scaling up from rodent studies [32,40]. However, the proportion of olfactory ensheathing cells, estimated by counting the number of p75-positive cells, varied from 48 to 76%. This can be attributed to various causes. First, some dogs provided a high proportion of olfactory ensheathing cells and some others provided much fewer despite undergoing the same biopsy method. In humans, it has been reported that age might alter olfactory ensheathing cell yield, with younger patients providing higher proportions of olfactory ensheathing cell [31]. In our study, there was not a significant correlation between olfactory ensheathing cell yield and age, or body weight, but other factors, including breed of dog and shape of skull, might also affect the cell proportion. Indeed, the shape of the skull might affect the size of the olfactory mucosa and might, in turn, affect mucosal layer anatomy and morphology. In humans, the precise site of biopsy of olfactory mucosa also influenced olfactory ensheathing cell yield [31,55]. For instance, septal olfactory mucosa yielded fewer olfactory ensheathing cells compared to the mucosa of the lateral nasal cavity or superior turbinate. Various dog breeds were included in this study. In each breed, the shape and size of the skull and frontal sinus are different, including brachycephalic and dolicocephalic dogs, which makes it difficult to obtain olfactory mucosa from equivalent anatomical regions. The dogs from which we obtained biopsies varied between methods, because most were recruited to a clinical trial and so could only undergo one method of biopsy. One possible explanation of the variable outcomes between techniques is that, through chance alone, the individuals that underwent rhinotomy were systematically less likely to yield a high proportion of olfactory ensheathing cells. As shown in Table 1, compared with other techniques a higher proportion of dogs undergoing rhinotomy were Dachshunds and so a possible explanation is that this breed is inherently a poor source of p75+ cells. On the other hand, this appears unlikely since the proportion of p75+ cells derived from rhinotomy samples in Dachshunds varied widely (from 0–88%), suggesting that it is more likely that the technique itself less reliably yields OECs than it being a breed-related problem. Furthermore, other factors we investigated (age, bodyweight and sex), which have previously been suggested to influence cell culture characteristics, did not appear to have strong effects on the characteristics of the isolated cell populations. Secondly, it is possible that there might have been differences between cultures in the number of remaining fibroblasts after the serum-free culture step. Fibroblasts in mucosal cultures are likely to be olfactory nerve fibroblasts. In our previous study, canine olfactory nerve fibroblasts proliferate rapidly in serum-containing medium [29], therefore, in some cultures, the olfactory nerve fibroblasts could not be excluded efficiently through serum-free purification methods and proliferated rapidly. Finally, we did not pre-specify an ‘ideal’ quantity of olfactory mucosa (either by weight or size) to harvest from these dogs and therefore this could have played an important role in the cell culture results.

The optimal proportion of mucosal olfactory ensheathing cell to promote axonal regeneration and improve functions following SCI is currently unknown and variable proportions of mucosal olfactory ensheathing cell have been used in rodent models [34,35,56–60]. A recent report described that higher proportions of olfactory mucosal ensheathing cells tended to lead to successful functional recovery after transplantation, while lower proportions (~10%) did not [57]. However, it has also been reported that low proportion of olfactory mucosal ensheathing cells (as low as 5% of the transplantation population) restored paw reaching function without axon regrowth across the lesion [60]. Furthermore, although olfactory mucosal ensheathing cells culture methods generally aim to produce transplant cell populations with a high proportion of p75-positive cells, it has also been suggested that contaminating olfactory nerve fibroblasts might support the promotion of axon regeneration by mucosal olfactory ensheathing cells [8,61–63]. In companion dogs, we have reported that autologous intraspinal transplantation of olfactory mucosal ensheathing cells was associated with improvement of locomotor function in chronic clinical SCI [40]. The data presented in the current report are derived from the transplanted cells used in that clinical trial. These cell populations were obtained by rhinotomy or the keyhole approach, and contained varying proportions and populations of olfactory mucosal ensheathing cells. However, similarly to rodent studies, there was no correlation between the proportion of p75-positive cells and functional outcome.

There is a debate on the exact nature of p75-positive cells—whether they represent a population of Schwann cells or are truly olfactory ensheathing cells—because the p75 label also detects Schwann cell, potentially associated with blood vessels in the olfactory mucosa biopsy. Currently, there is no specific marker for olfactory ensheathing cells. Indeed, olfactory ensheathing cells were originally thought to be olfactory nerve Schwann cells because of their similar morphology, expression of cell markers including p75, GFAP and S100β, and expression of several trophic factors [64–66]. It has also been reported that several markers including calponin, smooth actin protein and, more recently, stimulator of chondrogenesis-1 might recognize olfactory ensheathing cells but not Schwann cells [67–69]. However, there is controversy regarding the use of these markers as the gold standard to identify olfactory ensheathing cells because the expression of antigens were dependent of the purity of cell culture, origin of cell and environment of cell cultures [68, 70]. In fact, calponin staining detected olfactory mucosal fibroblasts rather than olfactory ensheathing cells [70], and smooth actin protein antibodies also recognized fibroblasts [68]. Therefore, these antigens are shared with contaminating cells in olfactory mucosal cell culture, and not widely accepted as markers for detection of olfactory ensheathing cells. For Schwann cells specifically, HNK-1 might have potential to identify Schwann cells in humans, rodents and dogs because it has been reported that the antigen was expressed by Schwann cells but not olfactory ensheathing cells [71–73]. Again however, there is a controversy regarding use of HNK-1 as a Schwann cell marker because the expression varies between species and culture methods. In fact, it has been reported that canine Schwann cells do not express HNK-1 *in vitro* [73, 74]. More recently, it has been suggested that use of aquaporin-1 antibody could label Schwann cells obtained from human sciatic nerve [69], but no direct comparison has been made with the cells from the olfactory mucosa, and therefore the marker has not been widely accepted.

Although difficulties persist with identification of olfactory ensheathing cells, we have, like others, made the assumption in our study that p75-positive cells would predominantly be olfactory ensheathing cells because of their preponderance in the olfactory mucosal biopsy sites. This is an approach shared by others for what is coined as ‘therapeutic transplants’ [75]. In the future, it might be feasible to further characterise canine OECs with flow-cytometry, following work from Kueh et al. [76]. Nonetheless, in the absence of a specific marker for olfactory ensheathing cells we cannot exclude the possibility of contamination with Schwann cells (in particular from blood vessels missed during the dissection of the biopsies).

## Conclusion

The olfactory mucosa obtained from dogs using the three different methods described here can each yield sufficiently large populations of olfactory ensheathing cells without severe adverse effects for the animal. These methods are directly applicable to clinical trials using the naturally-occurring canine SCI model [77] and mucosal olfactory ensheathing cells as part of the treatment strategies for spinal cord repair.

## Supporting information

S1 Appendix Cell proportion and phenotypic characteristics in cultures obtained from 21 Dachshunds by rhinotomy. (DOCX)
