# Supplementary material for: Methods of olfactory ensheathing cell harvesting from the olfactory mucosa in dogs
Source: PLoS One. 2019 Mar 6;14(3):e0213252. doi: 10.1371/journal.pone.0213252 (PMC6402693; doi:10.1371/journal.pone.0213252)
Supplement: S1 Appendix — (DOCX) [file pone.0213252.s001.docx]

| Case Number | Total cell number (x10^6^) | Proportion of Immunostaining Characteristic (%) | | |
| --- | --- | --- | --- | --- |
|  |  | p75+ | Fibronectin+ | Unidentified |
| Dog 1 | 7.5 | 7.7 | 91 | 1.3 |
| Dog 2 | 8.5 | 46.3 | 53.5 | 0.1 |
| Dog 3 | 8 | 66.1 | 27.8 | 6.2 |
| Dog 4 | 7.4 | 20.3 | 76.4 | 3.3 |
| Dog 5 | 5 | 51.7 | 46.6 | 1.7 |
| Dog 6 | 3.6 | 83.5 | 11.5 | 4.9 |
| Dog 7 | 8.6 | 82.4 | 17.6 | 0 |
| Dog 8 | 5.2 | 56.6 | 43.4 | 0 |
| Dog 9 | 7.3 | 80.1 | 19.6 | 0.3 |
| Dog 10 | 5 | 76.4 | 22.7 | 0.8 |
| Dog 11 | 7.4 | 0 | 100 | 0 |
| Dog 12 | 8.1 | 44.1 | 45.4 | 10.5 |
| Dog 13 | 5 | 41.9 | 57.7 | 0.4 |
| Dog 14 | 7.5 | 81.3 | 13.5 | 5.2 |
| Dog 15 | 2.1 | 6 | 90.6 | 3.4 |
| Dog 16 | 3.6 | 45.6 | 54.4 | 0 |
| Dog 17 | 9.3 | 88.5 | 11.5 | 0 |
| Dog 18 | 7.5 | 44.4 | 55.6 | 0 |
| Dog 19 | 6.2 | 17.4 | 82.6 | 0 |
| Dog 20 | 8.4 | 59.5 | 11.3 | 29.2 |
| Dog 21 | 8 | 75.4 | 17.9 | 6.7 |

**S1 Appendix.** Cell Proportion and Phenotypic Characteristics in Cultures Obtained from 21 Dachshunds by Rhinotomy
